# Hydrogen peroxide-independent production of *α*-alkenes by OleT_JE_ P450 fatty acid decarboxylase

**DOI:** 10.1186/1754-6834-7-28

**Published:** 2014-02-24

**Authors:** Yi Liu, Cong Wang, Jinyong Yan, Wei Zhang, Wenna Guan, Xuefeng Lu, Shengying Li

**Affiliations:** 1 Key Laboratory of Biofuels, Shandong Provincial Key Laboratory of Energy Genetics, Qingdao Institute of Bioenergy and Bioprocess Technology, Chinese Academy of Sciences, No. 189 Songling Road, Qingdao, Shandong 266101, China; 2 University of Chinese Academy of Sciences, Beijing 100049, China

**Keywords:** Alkenes, Biofuels, Monooxygenase, P450 fatty acid decarboxylase, Peroxygenase

## Abstract

**Background:**

Cytochrome P450 OleT_JE_ from *Jeotgalicoccus* sp. ATCC 8456, a new member of the CYP152 peroxygenase family, was recently found to catalyze the unusual decarboxylation of long-chain fatty acids to form *α*-alkenes using H_2_O_2_ as the sole electron and oxygen donor. Because aliphatic *α*-alkenes are important chemicals that can be used as biofuels to replace fossil fuels, or for making lubricants, polymers and detergents, studies on OleT_JE_ fatty acid decarboxylase are significant and may lead to commercial production of biogenic *α*-alkenes in the future, which are renewable and more environmentally friendly than petroleum-derived equivalents.

**Results:**

We report the H_2_O_2_-independent activity of OleT_JE_ for the first time. In the presence of NADPH and O_2_, this P450 enzyme efficiently decarboxylates long-chain fatty acids (C_12_ to C_20_) *in vitro* when partnering with either the fused P450 reductase domain RhFRED from *Rhodococcus* sp. or the separate flavodoxin/flavodoxin reductase from *Escherichia coli. In vivo*, expression of OleT_JE_ or OleT_JE_-RhFRED in different *E. coli* strains overproducing free fatty acids resulted in production of variant levels of multiple *α*-alkenes, with a highest total hydrocarbon titer of 97.6 mg·l^-1^.

**Conclusions:**

The discovery of the H_2_O_2_-independent activity of OleT_JE_ not only raises a number of fundamental questions on the monooxygenase-like mechanism of this peroxygenase, but also will direct the future metabolic engineering work toward improvement of O_2_/redox partner(s)/NADPH for overproduction of *α*-alkenes by OleT_JE_.

## Background

The urgency to develop sustainable fossil fuel alternatives is driven by rapidly increasing global consumption, irreversibly diminishing reserves, unpredictable geopolitical factors, fluctuating price of crude oil, growing concerns about national energy security, and serious environmental concerns surrounding immense greenhouse gas emissions mainly resulting from combustion of fossil fuels [1-3]. Biofuels produced from biological resources represent a compelling alternative to fossil fuels because they are renewable and more environmentally friendly [4-7]. Among various biofuels, bioethanol and biodiesel are dominating the current global market. However, it is widely accepted that the ideal biofuels are bio-hydrocarbons, especially the medium- to long-chain fatty alkanes or alkenes, because they highly mimic the chemical composition and physical characteristics of petroleum-based fuels [8-10]. Thus, the biosynthetic pathways for aliphatic hydrocarbons from diverse organisms have been attracting great attentions in recent years [11-13]. Particularly, the scalable and cost-effective microbial biosynthesis of fatty alkanes or alkenes is considered as one of the most promising ways to produce ‘drop-in compatible’ biofuels [5,8].

To date, four microbial biosynthetic pathways that convert free fatty acids or fatty acid thioesters into bio-hydrocarbons have been identified, including the cyanobacterial pathways consisting of an acyl-acyl carrier protein (acyl-ACP) reductase and an aldehyde decarbonylase, which together convert fatty acyl-ACPs into alkanes [14]; a three-gene cluster responsible for generating alkenes with internal double bonds through the head-to-head condensation of two fatty acyl-coenzyme A (acyl-CoA) molecules in *Micrococcus luteus*[15]; a unique P450 decarboxylase OleT_JE_ from *Jeotgalicoccus* sp. ATCC 8456, which directly decarboxylates long-chain fatty acids to form *α*-olefins in presence of H_2_O_2_[16,17]; and a type I polyketide synthase from *Synechococcus* sp. PCC 7002 [18,19], which is capable of transforming fatty acyl-ACPs into *α*-olefins via sequential polyketide synthase chain elongation, keto reduction, sulfonation mediated by its sulfotransferase domain, and the coupled hydrolysis and decarboxylation catalyzed by the thioesterase domain.

Among these pathways, the single-step decarboxylation of fatty acids catalyzed by OleT_JE_ P450 enzyme [16] apparently represents the simplest one. Moreover, it directly uses free fatty acids instead of fatty acid thioesters as substrates, which is believed to be advantageous for metabolic engineering because fatty acids are more abundant and their abundance and composition can be well manipulated in *E. coli*[20-22], one of the most developed microbial cell factories. Thus, this P450 fatty acid decarboxylative machinery may hold great potential to be engineered into a biological *α*-alkene-producing system. Notably, in addition to being biofuel molecules, *α*-alkenes are also used broadly for making lubricants, polymers and detergents [17,23,24].

The superfamily of cytochrome P450 enzymes (CYPs) are considered among the most versatile biocatalysts in nature [25]. Typically, P450 enzymes employ one or more redox partner proteins to transfer two electrons from NAD(P)H to the heme iron reactive center for dioxygen activation, and then insert one atom of O_2_ into their substrates [25-27]. Therefore, these oxidative biocatalysts are often termed as P450 monooxygenases. However, the CYP152 family members such as P450_SPα_[28], P450_BSβ_[29] and OleT_JE_[16] have been identified to exclusively use H_2_O_2_ as the sole electron and oxygen donor, and are thus classified into a unique P450 peroxygenase category.

Practically, the peroxygenase activity of P450 enzymes is often treated as an advantageous feature because H_2_O_2_ is much cheaper than NADPH and redox proteins in many P450 applications such as biocatalysts for *in vitro* synthetic reactions and enzyme additives in laundry detergents [30,31]. However, the large-scale production of low-cost *α*-alkene biofuels by OleT_JE_ P450 decarboxylase cannot rely on the H_2_O_2_-dependent enzymatic system because the use of large amounts of peroxide is cost prohibitive, and high concentration of H_2_O_2_ can quickly deactivate biocatalysts [32].

Therefore, the H_2_O_2_-independent activity of OleT_JE_, if it exists, is preferred for cost-effective microbial production of *α*-alkenes. In the present study, we demonstrated such activity for the first time. Through engineering a self-sufficient version of OleT_JE_ by fusing it to the *Rhodococcus* fusion reductase (RhFRED) domain from *Rhodococcus* sp. NCIMB 9784 [33], the catalytic activity of the resultant fusion P450 enzyme can be solely driven by NADPH. We also found that *E. coli* flavodoxin (Fld) and flavodoxin reductase (FdR) are capable of supporting OleT_JE_ activity as well. Guided by these new findings, our initial metabolic engineering efforts based on the H_2_O_2_-independent decarboxylation of fatty acids by OleT_JE_*in vivo* led to a group of *α*-alkene overproducers with the best one producing 97.6 mg·l^-1^ total *α*-alkenes.

## Results

### *In vitro* fatty acid decarboxylation by OleT_JE_ and OleT_JE_-RhFRED

**Figure 1:**
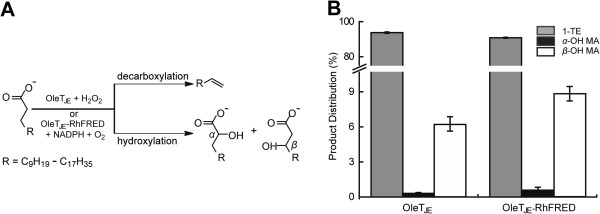
**Decarboxylation and hydroxylation reactions catalyzed by OleT**_**JE. **_**(A)** The decarboxylation and hydroxylation of fatty acids (C_12_ to C_20_) catalyzed by OleT_JE_. **(B)** Product distribution of the decarboxylation and hydroxylation reactions catalyzed by purified OleT_JE_ and OleT_JE_-RhFRED, respectively. Myristic acid was used as substrate. 1-TE, 1-tridecene; *α*-OH MA, *α*-hydroxy myristic acid; *β*-OH MA, *β*-hydroxy myristic acid.

The unusual activity of the first P450 fatty acid decarboxylase OleT_JE_ was determined by reconstituted *in vitro* reactions [16]. Specifically, OleT_JE_ was able to decarboxylate stearic acid and palmitic acid to generate 1-heptadecene and 1-pentadecene, respectively, in the presence of H_2_O_2_ (Figure 1A). It also catalyzed the *α*- and *β*-hydroxylation of fatty acids as side reactions. Using the purified P450 enzymes (Additional file 1: Figure S1 and Additional file 2: Figure S2), we first confirmed these three types of activity of OleT_JE_ using myristic acid (C_14_) as substrate. Consistent with the previous report [16], fatty acid decarboxylation was the dominant reaction, while the *α*- and *β*-hydroxymyristic acid only accounted for 0.2% and 6.1% of total products, respectively (Figure 1B).

According to previous amino acid sequence analysis, OleT_JE_ was assigned to the CYP152 family, with the well-studied P450_BSβ_[29,34,35] from *Bacillus subtilis* and P450_SPα_[28,36] from *Sphingomonas paucimobilis* as family members. Essentially, this family of P450 enzymes utilizes H_2_O_2_ as the sole electron and oxygen donor to oxidize their substrates, in contrast to most other P450 monooxygenases, whose catalytic activity depends on dioxygen, NAD(P)H and redox partner protein(s) [37]. Thus, these P450 enzymes are functionally referred to as peroxygenases [38]. Indeed, it was reported that P450 reductase systems such as ferredoxin and ferredoxin reductase did not support the activity of P450_BSβ_ and P450_SPα_[36,39]. This is consistent with the substitution of Pro^246^ and Arg^245^ (numbering in OleT_JE_) in CYP152 peroxygenases for the highly conserved threonine and an acidic residue (for example, Glu or Asp) in most P450 monooxygenases (Additional file 3: Figure S3), which are involved in a specific H-bond network responsible for delivery of protons during the P450 catalytic cycle [34,40].

However, in the study of another CYP152-member P450_CLA_, from the anaerobic microorganism *Clostridium acetobutylicum*[41], it was demonstrated that both P450_CLA_ and P450_BSβ_ functioned when provided with O_2_, NADPH and flavodoxin/flavodoxin reductase from *E. coli* or the diflavin reductase domain of P450_BM3_ from *Bacillus megaterium*, suggesting an alternative monooxygenase-like mechanism of these P450 peroxygenases.

**Figure 2:**
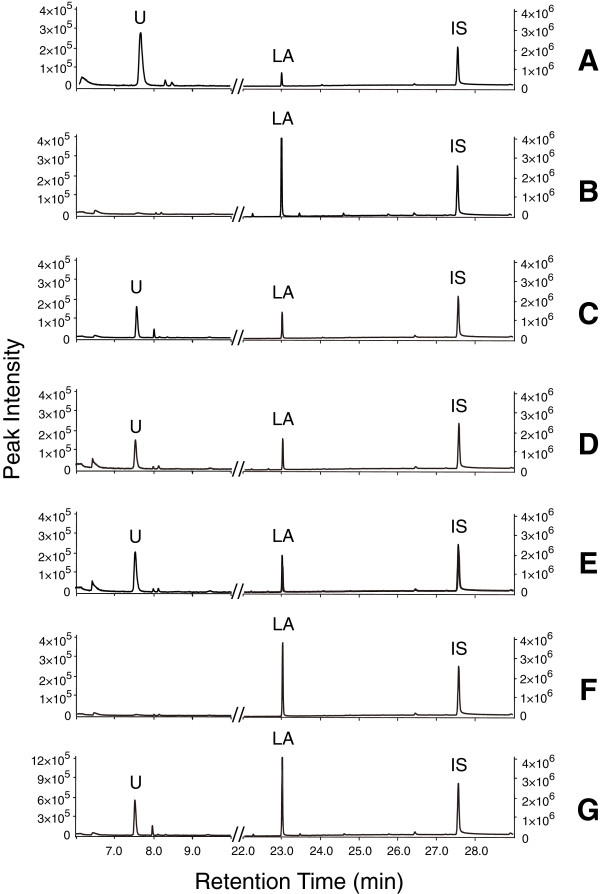
**Gas chromatography-mass spectroscopy analysis of decarboxylation reactions (10 min) catalyzed by OleT**_**JE **_**or OleT**_**JE**_**-RhFRED under different reaction systems. (A)** OleT_JE_ + H_2_O_2_; **(B)** OleT_JE_ + NADPH; **(C)** OleT_JE_-RhFRED + NADPH; **(D)** OleT_JE_-RhFRED + H_2_O_2;_**(E)** OleT_JE_-RhFRED + NADPH + H_2_O_2;_**(F)** OleT_JE_-RhFRED in absence of NADPH; **(G)** authentic standards of 1-undecene (U) lauric acid (LA) and heptadecanoic acid (IS: internal standard).

To investigate whether OleT_JE_ peroxygenase can also function as a monooxygenase like P450_CLA_ and P450_BSβ_, the RhFRED reductase domain from *Rhodococcus* sp. NCIMB 9784 [33] was fused to the C-terminus of OleT_JE_. In the presence of the electron donor NADPH, the fusion protein OleT_JE_-RhFRED successfully converted lauric acid (C_12_) into 1-undecene with the conversion ratio of 51.1 ±0.3%, approximately half that of OleT_JE_ plus H_2_O_2_ (93.0 ±4.3% conversion; Figure 2). Notably, the product distribution (decarboxylation *versus* hydroxylation) of the OleT_JE_-RhFRED catalyzed reaction was similar to that of the OleT_JE_ reaction (Figure 1B), indicating the fused reductase domain has no significant impact on the catalytic mechanism of P450 domain. As control, OleT_JE_ cannot directly use NADPH as electron donor. OleT_JE_-RhFRED was not active by itself. Interestingly, OleT_JE_-RhFRED retained its ability to use H_2_O_2_ as a cofactor, but there was not a significant additive effect between NADPH and H_2_O_2_ for supporting the activity of OleT_JE_-RhFRED (Figure 2).

**Figure 3:**
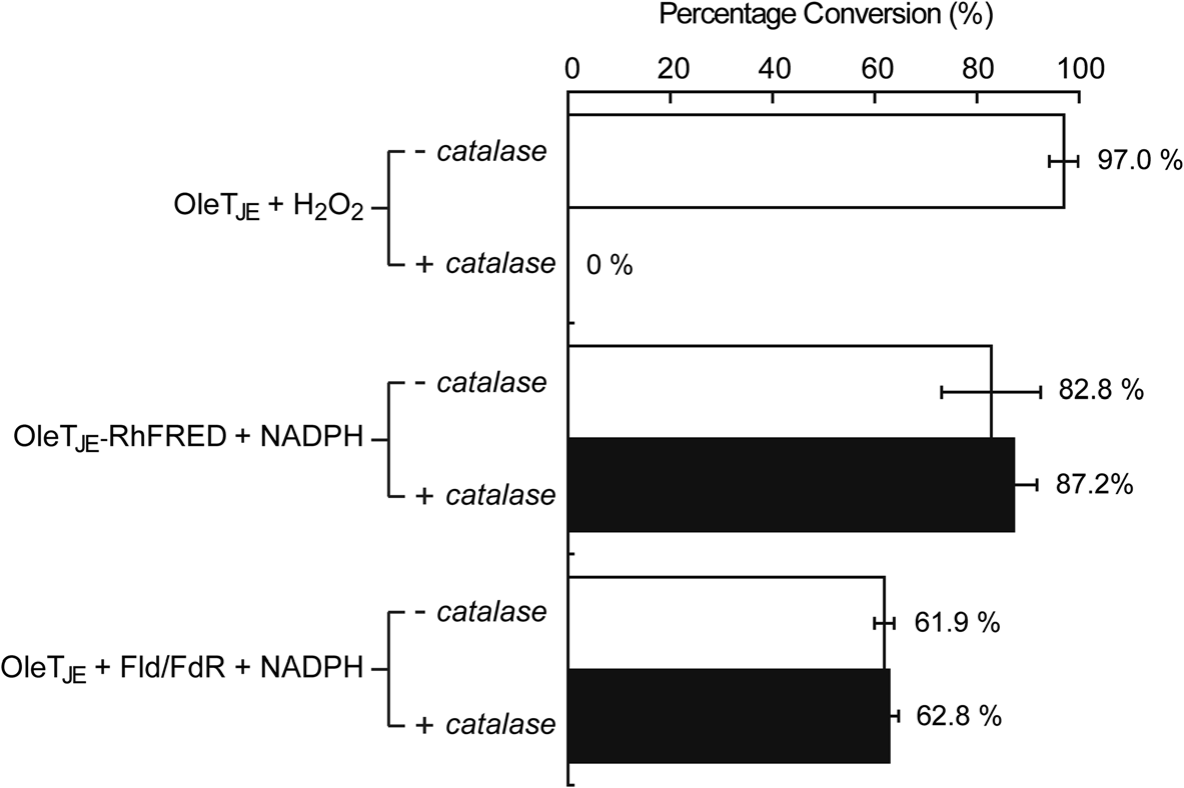
**Effects of catalase on the *in vitro *decarboxylation activity of three OleT**_**JE **_**(1 μM) reaction systems. Systems comprised OleT**_**JE **_**plus H**_**2**_**O**_**2**_**, OleT**_**JE**_**-RhFRED plus NADPH, and OleT**_**JE **_**plus flavodoxin and flavodoxin reductase plus NADPH.** The percentage conversion of myristic acid is shown beside each bar.

To prevent the spontaneous generation of H_2_O_2_ during the OleT_JE_-RhFRED reaction, which could complicate interpretation of the results, dithiothreitol (DTT: the reducing agent often used in the storage and reaction buffer of P450 proteins) was omitted from all buffers used during protein purification, storage and reaction because it has previously been reported that DTT, dioxygen and the heme iron center of P450 can react to generate H_2_O_2_[16,42]. To further exclude the possibility that H_2_O_2_ could be produced from the peroxide shunt pathway during the monooxygenase catalytic cycle [27], bovine liver catalase that is able to efficiently eliminate H_2_O_2_ was added to the fatty acid decarboxylation reactions (in the DTT-free buffer) catalyzed by OleT_JE_ and OleT_JE_-RhFRED. In the OleT_JE_ plus H_2_O_2_ system, pre-addition of catalase completely abolished the reaction (Figure 3). By contrast, in the OleT_JE_-RhFRED plus NADPH reaction, pre-added catalase did not significantly change (indeed, it slightly improved) the conversion of myristic acid to 1-tridecene (Figure 3). This clearly indicates that the decarboxylation activity of OleT_JE_-RhFRED can be solely supported by NADPH.

### Substrate specificity of OleT_JE_ and OleT_JE_-RhFRED

**Figure 4:**
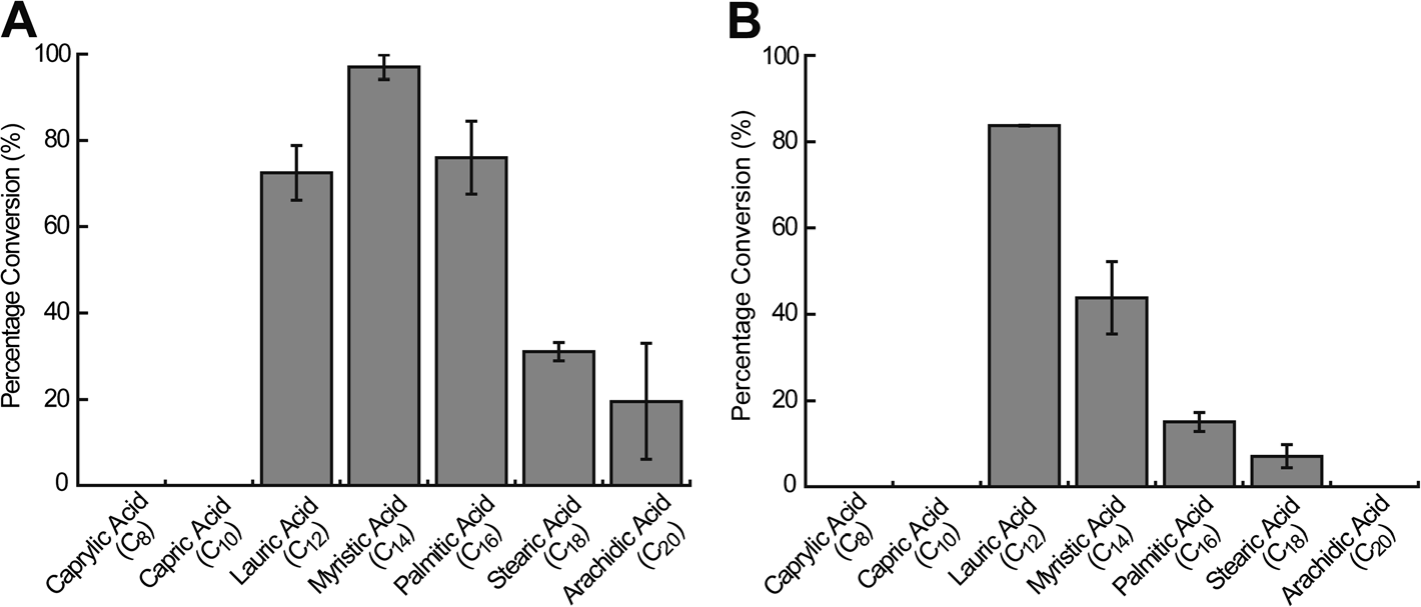
**Substrate preference spectrum of (A) OleT**_**JE **_**and (B) OleT**_**JE**_**-RhFRED.** The substrate preference was determined by calculating the percentage conversion of each fatty acid substrate into corresponding *α*-alkene product. In these assays, 0.2 μM enzymes were used.

The substrate preference of OleT_JE_ and OleT_JE_-RhFRED provides useful information to help understand the *in vivo* behavior of these fatty acid decarboxylases and to guide the metabolic engineering of fatty acid biosynthesis. Thus, we determined their substrate specificity (Figure 4) using a number of straight-chain saturated fatty acids with even-numbered chain length ranging from C_8_ to C_20_. In the case of OleT_JE_ (Figure 4A), myristic acid (C_14_) turned out to be the best substrate for olefin production among the tested fatty acids with the conversion ratio of 97.0 ± 2.8%. Lauric acid (C_12_) and palmitic acid (C_16_) were sub-optimal. OleT_JE_ was only able to convert a minority of stearic acid (C_18_) and arachidic acid (C_20_), but could not decarboxylate capric acid (C_10_) or caprylic acid (C_8_).

In comparison, the substrate preference spectrum of OleT_JE_-RhFRED slightly shifted toward shorter fatty acids (Figure 4B), which suggests that the P450-reductase interaction might induce small conformational change of the OleT_JE_ active site. Specifically, OleT_JE_-RhFRED showed the highest activity against lauric acid (83.8 ±0.1% conversion), but was inactive toward arachidic acid. Similar to OleT_JE_, OleT_JE_-RhFRED could not decarboxylate capric acid or caprylic acid. It is also worth noting that the system of OleT_JE_-RhFRED plus NADPH plus O_2_ was less active than OleT_JE_ plus H_2_O_2_ when reacting with most tested fatty acids except for lauric acid, suggesting OleT_JE_ might have evolved to become a better peroxygenase than a monooxygenase.

### *In vivo* production of *α*-alkenes in *Escherichia coli*

Motivated by the efficient *in vitro* conversion of free fatty acids (C_n_) to corresponding *α*-alkenes (C_n-1_) by two related but mechanistically distinct decarboxylation systems including OleT_JE_ plus H_2_O_2_ and OleT_JE_-RhFRED plus NADPH plus O_2_, we sought to engineer hydrocarbon-producing strains of *E. coli*. To guarantee sufficient substrate supply of free fatty acids for OleT_JE_ P450 decarboxylase, two previously engineered fatty acid-overproducing strains including XL100 (BL21:*ΔfadD*) and XL100/(pMSD8 + pMSD15) [43] were used as hosts for the decarboxylases. Transformation of these two strains with pET28b-*oleT*_*JE*_ or pET28b-*oleT*_*JE*_-*RhFRED* resulted in YL5 (XL100 with pET28b-*oleT*_*JE*_), YL6 (XL100 with pET28b-*oleT*_*JE*_-*RhFRED*), YL7 (XL100 with pET28b-*oleT*_*JE*_, pMSD8 and pMSD15), and YL8 (XL100 with pET28b-*oleT*_*JE*_-*RhFRED*, pMSD8 and pMSD15) (Table 1).

**Table 1:** Strains and plasmids used in this study

**Strain or plasmid**	**Relevant characteristics**	**Source or reference**
*E. coli* strains		
BL21(DE3)	F^-^*ompT gal dcm lon hsdSB* (r_B_^-^ m_B_^-^) λ(DE3)	Novagen
DH5α	F^-^ ξ80*lacZ*ΔM15 Δ (Δl*acZYA*-*argF*) U169 *recA*1 *endA*1 *hsdR*17 (rk^-^, mk^+^) *phoA supE*44 *thi-*1 *gyrA96 relA1 λ*^-^	Invitrogen
XL100	BL21: Δ*fadD*^a^	[43]
YL1	BL21(DE3) with pET28b-*oleT*_*JE*_	This study
YL2	BL21(DE3) with pET28b-*oleT*_*JE*_-*RhFRED*	This study
YL3	BL21(DE3) with pACYCDuet-*fdR*	This study
YL4	BL21(DE3) with pCDEDuet-*fld*	This study
YL5	XL100 with pET28b-*oleT*_*JE*_	This study
YL6	XL100 with pET28b-*oleT*_*JE*_-*RhFRED*	This study
YL7	XL100 with pET28b-*oleT*_*JE*_, pMSD8^b^, pMSD15^c^	This study
YL8	XL100 with pET28b-*oleT*_*JE*_-*RhFRED*, pMSD8, pMSD15	This study
*Jeotgalicoccus sp*. ATCC 8456	Wild type	ATCC
Plasmids		
pET28b	Km^r^, T7 promoter, pBR322 origin	Novagen
pET28b-*oleT*_*JE*_	Km^r^, pET28b derivative containing *oleT*_*JE*_ gene	This study
pET28b-*oleT*_*JE*_-*RhFRED*	Km^r^, pET28b derivative containing *oleT*_*JE*_ and *RhFRED* gene	This study
pACYCDuet-1	Cm^r^, T7 promoter, P15A origin	Novagen
pACYCDuet-*fdR*	Cm^r^, pACYCDuet-1 derivative containing *fdR* gene	This study
pCDFDuet-1	Str^r^, T7 promoter, CloDF13 origin	Novagen
pCDEDuet-*fld*	Str^r^, pCDFDuet-1 derivative containing *fld* gene	This study
pMSD8	Amp^r^, *P*_*T7*_: *accB*, *C*, *D*, *A,* pSC101 origin	[43]
pMSD15	Cm^r^, P_*BAD*_: *tesA,* p15a origin	[43]

^a^The *fadD* gene encodes the acyl-CoA synthetase, which is the first enzyme involved in fatty acid degradation. ^b^pMSD8: the expression vector for *E. coli* acyl-CoA carboxylase. ^c^pMSD15: the plasmid used for overexpression of the *E. coli* thioesterase gene *tesA*. Amp, ampicillin; Cm, chloramphenicol; Km, kanamycin; Str, streptomycin.

**Figure 5:**
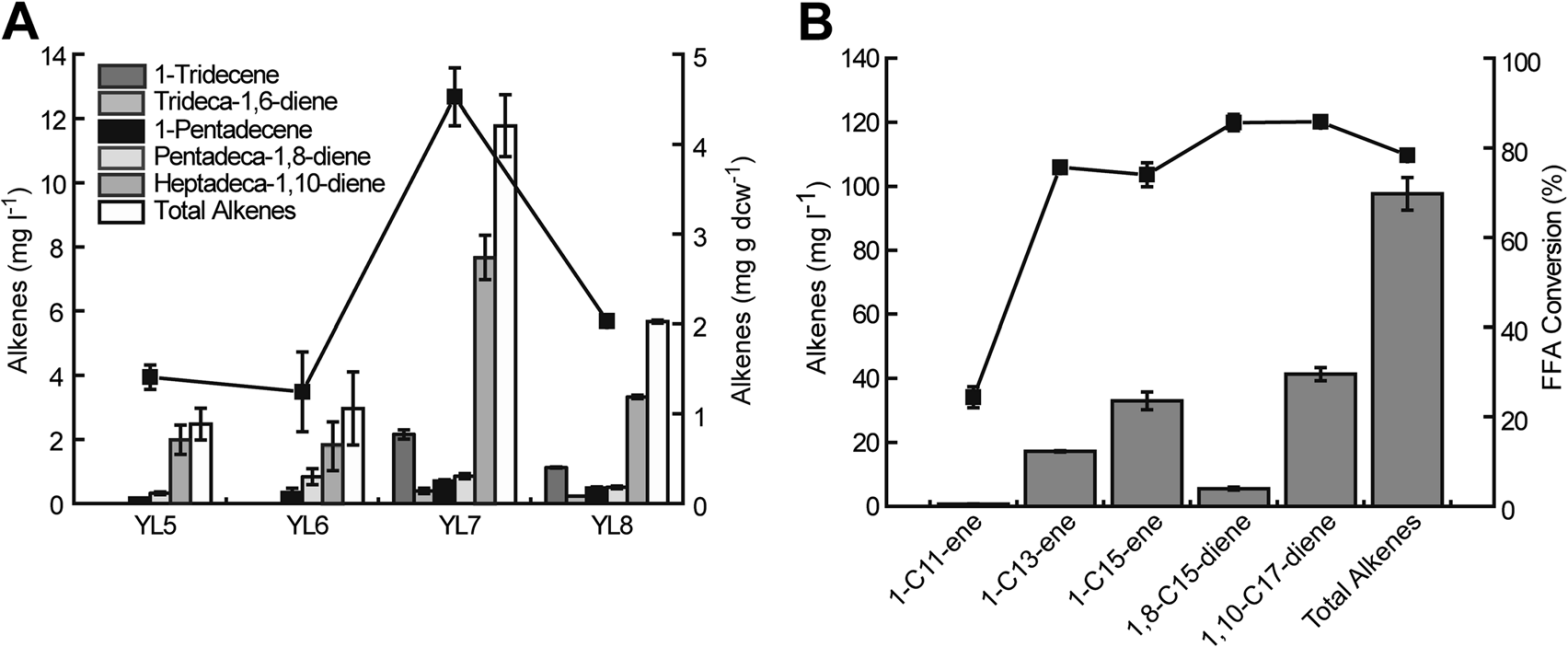
**Production of *α*-alkenes by heterologous expression of OleT**_**JE **_**or OleT**_**JE**_**-RhFRED in fatty acid-overproducing *Escherichia coli *strains. (A)** Production of *α*-alkenes by OleT_JE_ and OleT_JE_-RhFRED in two fatty acid-overproducing *E. coli* strains. The line chart denotes the alkene productivity. **(B)** The *α*-alkene production and fatty acid conversion profiles of strain YL7 in the defined mineral medium. The histograms demonstrate the titer of each detected *α*-alkene titers. The line chart demonstrates the conversion ratio of corresponding fatty acids. FFA, free fatty acid.

As expected, all these engineered strains successfully accumulated multiple *α*-alkenes, including 1-tridecene, trideca-1,6-diene, 1-pentadecene, pentadecene-1,8-diene and heptadeca-1,10-diene (Figure 5A and Additional file 4: Figure S4), which correspond to the decarboxylation products of the 14:0, 14:1, 16:0, 16:1 and 18:1 fatty acids, respectively. This fatty acid composition is consistent with previous reports [43]. In all cases, the major alkene product was heptadeca-1,10-diene with the highest yield of 7.7 mg·l^-1^ in YL7. Since XL100/(pMSD8 + pMSD15) is able to produce more free fatty acids than XL100 [43], the total alkene titers of YL7 and YL8 were significantly greater than those of YL5 and YL6. Notably, expression of P450 decarboxylases did not significantly affect the cell growth of all strains (Additional file 5: Figure S5).

According to a previous report [44], H_2_O_2_ levels in growing *E. coli* are less than 20 nM, and 2 μM H_2_O_2_ could cause substantial growth inhibition. Therefore, the physiological concentration of H_2_O_2_ is far below the optimal H_2_O_2_ concentration (200 to 500 μM) [16] for supporting OleT_JE_ catalysis. Thus, we had predicted that OleT_JE_-RhFRED using endogenous NADPH, whose physiological concentration is around 120 μM [45], might catalyze the decarboxylation more efficiently than OleT_JE_ using H_2_O_2_ as a cofactor. Unexpectedly, the highest titer (11.8 mg·l^-1^) and productivity (4.5 mg·g dcw^-1^) of total alkenes was achieved by YL7 with OleT_JE_ instead of YL8 with OleT_JE_-RhFRED. This contradiction has led to a hypothesis that OleT_JE_ is able to employ the native redox system of *E. coli* to drive catalysis, such as P450_CLA_ and P450_BSβ_[41].

To test this hypothesis, we expressed and purified the *E. coli* Fld and FdR. In the *in vitro* reaction containing OleT_JE_, Fld, FdR and NADPH, myristic acid was substantially converted into 1-tridecene (Figure 3). Therefore, Fld and FdR probably better serve OleT_JE_ than the fused RhFRED *in vivo*, explaining why YL7 was a better alkene producer than YL8. However, the supportive role of H_2_O_2_ cannot be entirely neglected.

Inspired by these results, we attempted to enhance the level of dissolving dioxygen for improving OleT_JE_ catalysis during the cultivation of the best alkene producer, YL7, by applying a higher rotation rate of 250 rpm. Moreover, the defined mineral medium [14] containing 3% glucose was used to standardize the culture condition for the future metabolic engineering, and the culture time was extended to 40 h. Significantly, the total alkene titer of YL7 under these conditions was 97.6 mg·l^-1^ (Figure 5B), which is almost seven-fold higher than that in lysogeny broth (LB) medium at 220 rpm for 20 h (Figure 5A). The productivity of total alkenes was also improved from 4.5 mg·g dcw^-1^ (in LB over 20 h) to 24.9 mg·g dcw^-1^ (in the defined mineral medium over 40 h, data not shown). Again, heptadeca-1,10-diene was the major alkene product (41.4 mg·l^-1^). As control, the production of free fatty acids by the strain XL100/(pMSD8 + pMSD15) without OleT_JE_ expression was evaluated. Octadec-11-enoic acid was the most abundant fatty acid with a titer of 96.1 mg·l^-1^ (Additional file 6: Figure S6). This well explains why its decarboxylated product, heptadeca-1,10-diene, had the highest yield among produced alkenes (Figure 5). With respect to fatty acid conversion, octadec-11-enoic acid had the highest conversion ratio (85.9%) followed by hexadec-9-enoic acid (85.7%), myristic acid (75.7%), palmitic acid (74.0%) and lauric acid (24.4%). The low conversion of lauric acid seems to be inconsistent with the *in vitro* result (Figure 4A), which is likely due to the low intracellular concentration of the C_12_ fatty acid.

## Discussion

The cytochrome P450 enzymes are a superfamily of b-type heme proteins, capable of catalyzing more than 20 different types of reactions [25,46,47]. The H_2_O_2_-assisted decarboxylation of long-chain fatty acids catalyzed by P450 OleT_JE_ represents a novel activity of this highly versatile superfamily. This activity may be mechanistically similar to that of P450_Rm_ (CYP53B) from the yeast *Rhodotorula minuta*[48], which decarboxylates isovalerate to form isobutene.

**Figure 6:**
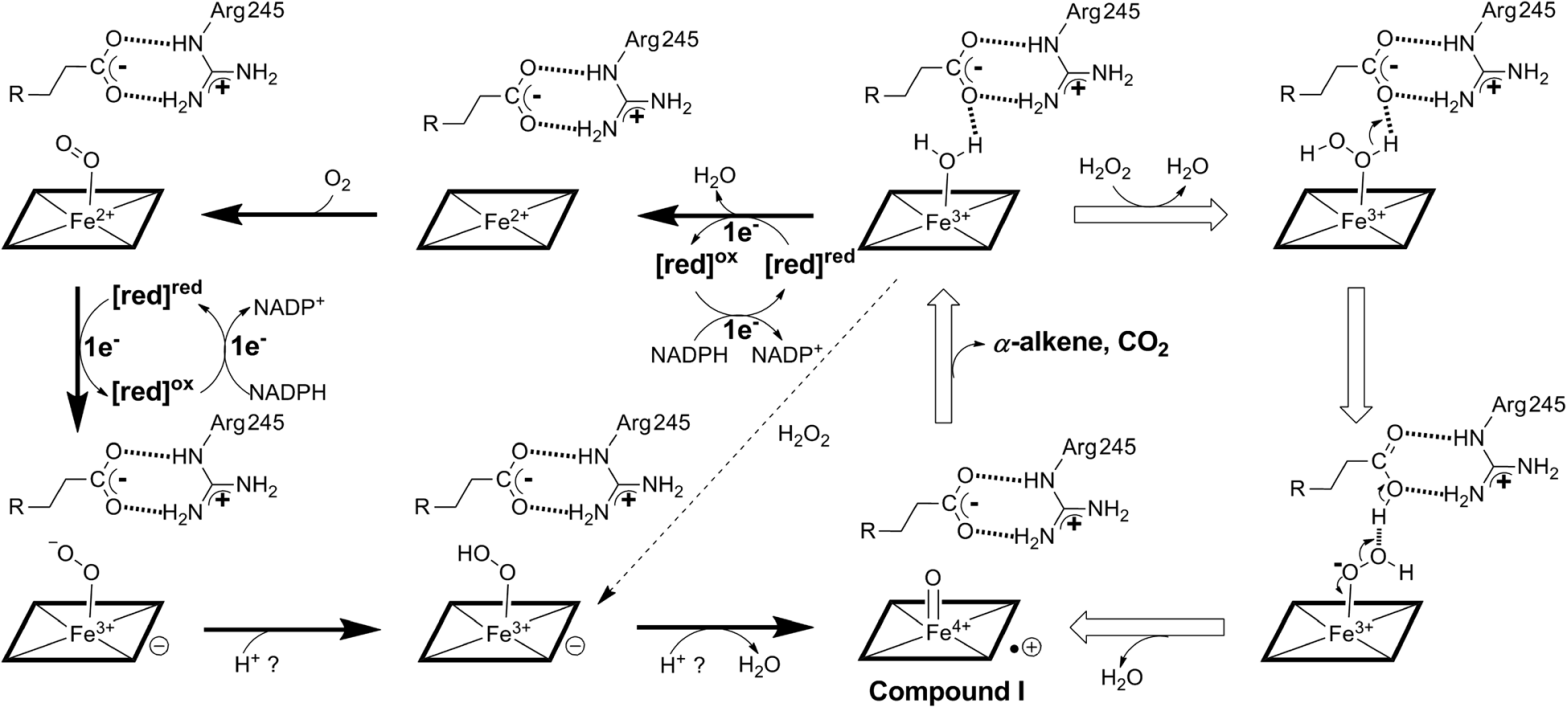
**Proposed two alternative catalytic mechanisms of OleT**_**JE **_**with (monooxygenase-like) or without (peroxygenase-like) redox systems.** The Arg^245^ is proposed to be required for substrate anchoring via the strong electrostatic interactions with the carboxyl group of fatty acid substrate. The supply of two protons in the putative monooxygenase catalytic cycle remains unclear. The dashed arrow indicates the peroxide shunt pathway.

According to protein sequence alignment (Additional file 3: Figure S3), OleT_JE_ belongs to the CYP152 peroxygenase family together with the well-studied P450_BSβ_ and P450_SPα_[28,29], and other members. Functionally, these P450 peroxygenases were previously thought to have no ability of utilizing dioxygen to drive catalysis as typical P450 monooxygenases do [39]. However, we have unambiguously demonstrated that OleT_JE_ can perform H_2_O_2_-independent catalysis when partnering with the fused RhFRED reductase or the *E. coli* Fld/FdR system to transfer electrons from NADPH to the heme iron reactive center. This strongly suggests that OleT_JE_ can undergo the monooxygenase catalytic cycle to generate the highly reactive ferryl-oxo cation radical species (Compound I) for catalysis. In the well-accepted mechanism for dioxygen activation in P450 monooxygenases (Figure 6), two protons (and two electrons from NADPH) are required for generation of Compound I [27]. However, the conserved threonine and an acidic residue involved in proton delivery in normal P450 monooxygenases [34,40] are replaced by Pro and Arg, respectively, which are absolutely conserved in OleT_JE_ and all other known CYP152 family members (Additional file 3: Figure S3). This strongly suggests an unknown proton transfer pathway. To elucidate this hypothetical pathway, we are currently seeking to solve the crystal structure of OleT_JE_.

Evolutionarily, because the early Earth’s environment probably had more H_2_O_2_ and peroxygenated organic chemicals than O_2_, P450 peroxygenases are presumed to have emerged ahead of P450 monooxygenases [38]. Thus, the ability of most P450 monooxygenases to use H_2_O_2_ as a surrogate for the O_2_/2e^-^/2H^+^ system (peroxide shunt pathway, Figure 6) could be understood as a remnant function inherited from their peroxygenase ancestors, whereas the OleT_JE_ P450 peroxygenase bearing the monooxygenase property might represent a transition species during the evolving process from peroxygenase to monooxygenase.

Under certain circumstances, such as *in vitro* synthetic reactions using purified P450s and application of P450 enzymes in laundry detergents, the peroxygenase activity supported by H_2_O_2_ is advantageous because expensive redox partner proteins and NAD(P)H are not needed [30,31]. However, the peroxygenase nature is not a good feature if attempting to construct a biofuel-producing microorganism by taking advantage of the fatty acid decarboxylation activity of OleT_JE_. Essentially, the intracellular level of H_2_O_2_ cannot be increased to the concentration (10^1^ to 10^2^ μM range) required for efficiently supporting OleT_JE_ because at this level, H_2_O_2_ is toxic or fatal to all organisms including *E. coli*. Thus, that the activity of P450 OleT_JE_ can be supported by O_2_/redox partner(s)/NADPH besides H_2_O_2_ is a significant discovery because the former three factors are more viable targets for metabolic engineering [49-51]. Being aware of this, future metabolic engineering work aiming to improve the *in vivo* productivity of *α*-alkenes by OleT_JE_ probably should be directed to improvement of the intracellular level of dioxygen, redox partner protein(s) and/or NADPH. For example, among numerous metabolic strategies for *in vivo* up-regulation of NADPH [49], the NADPH regenerating system could be used for maintaining the high intracellular level of this reducing cofactor to better support the activity of OleT_JE_ and the overproduction of fatty acids [52,53], both of which require a sufficient supply of NADPH.

In the mutant *E. coli* strains with up-regulated fatty acid biosynthesis (XL100 and XL100/(pMSD8 + pMSD15)), both OleT_JE_ and OleT_JE_-RhFRED were functional, therefore significantly converting fatty acids into *α*-alkenes. Consistent with the previous report [16], the common major product of four engineered alkene producers (YL5-8) turned out to be heptadeca-1,10-diene, but the second most abundant alkene varied (Figure 5A). This is likely because octadec-11-enoic acid (the precursor of heptadeca-1,10-diene) was the predominant component of the fatty acid pool of the tested strains (Additional file 6: Figure S6). However, there might be an additional reason that octadec-11-enoic acid is a preferred substrate *in vivo*. Interestingly, in previous studies [43,54,55], palmitic acid rather than octadec-11-enoic acid was the major component in the fatty acid pool of *E. coli*. This inconsistency could be due to different culture conditions.

After 20 h cultures of YL7 in LB medium, significant amounts of fatty acids remained unreacted with OleT_JE_ (Additional file 4: Figure S4). By contrast, the majority of fatty acids in YL7 were consumed by OleT_JE_ over the longer and more oxygenated cultivation in the defined mineral medium (Figure 5B). This suggests that, at the current stage, the yield of fatty acids might be the major limiting factor for further improvement of *α*-alkene titers. Thus, the overproduction of fatty acids needs to be significantly optimized prior to other engineering efforts on up-regulating the level of OleT_JE_, redox partners, O_2_ and NADPH.

In this report, the best bio-hydrocarbon-producing strain YL7 accumulated 97.6 mg·l^-1^ of total alkenes. This yield is comparable to or better than a majority of engineered alkane and alkene biosynthetic pathways with reported yields. These include the artificial alkane biosynthetic pathway in *E. coli* consisting of the carboxylic acid reductase from *Mycobacterium marinum* that catalyzes formation of fatty aldehydes directly from fatty acids, and the aldehyde decarbonylase from *Synechocystis* sp. PCC 6803 to produce alkanes (yield: approximately 2 mg·l^-1^) [56]; the hybrid system using the fatty acid reductase complex (LuxC, LuxE and LuxD) to provide fatty aldehyde as the substrate for downstream aldehyde decarbonylase to generate alkanes (yield: approximately 2 to 5 mg·l^-1^) [20]; and the ATP required decarboxylation of 3-hydroxy-3-methylbutyrate catalyzed by the R74H mutant of mevalonate diphosphate decarboxylase from *Saccharomyces cerevisiae* (productivity: 5,888 pmol·h^-1^·g cells^-1^) [57]. It is only lower than the 300 mg·l^-1^ of total alkane titer produced when the cyanobacterial pathway consisting of the acyl-ACP reductase Orf1594 from *Synechococcus elongates* PCC7942 and the aldehyde decarbonylase from *Nostoc punctiforme* PCC73102 was heterologously expressed in *E. coli*[14]. However, it is expected that more metabolic engineering efforts and optimization of fermentation will further increase the total alkene titers of the OleT_JE_/*E. coli* system, which is currently ongoing in our laboratory.

## Conclusions

The H_2_O_2_ independence of OleT_JE_ described in this work not only raises a number of fundamental questions regarding its monooxygenase-like mechanism, but also could direct future metabolic engineering work toward improvement of O_2_/redox partner(s)/NADPH for optimal activity of OleT_JE_*in vivo*. Considering its high conversion rate *in vitro* and H_2_O_2_-independent functionality *in vivo*, it is of great potential for engineering a hyper-producer of *α*-alkenes on the basis of OleT_JE_.

## Methods

### Materials

Fatty acid substrates, terminal alkene authentic standards, and derivatizing reagents were purchased from TCI (Shanghai, China). Antibiotics were obtained from SolarBio (Beijing, China). Other chemicals were from Sigma Aldrich (St. Louis, MO, USA) or Ameresco (Solon, OH, USA). Oligonucleotides were synthesized by Sangon Biotech (Shanghai, China), and their sequences are shown in Additional file 7: Table S1. The *Pfu* DNA polymerases and all restriction endonucleases were obtained from Fermentas (Vilnius, Lithuania) or Takara (Dalian, China). The kits used for molecular cloning were from OMEGA Bio-Tek (Jinan, China) or Promega (Madison, WI, USA). Protein purification used Qiagen Ni-NTA resin (Valencia, CA, USA), Millipore Amicon Ultra centrifugal fliters (Billerica, MA, USA) and PD-10 desalting columns from GE Healthcare (Piscataway, NJ, USA). Bovine liver catalase was purchased from Sigma Aldrich.

### Molecular cloning

Strains and plasmids constructed and used in this study are listed in Table 1. The gene of *oleT*_*JE*_ was amplified from the genomic DNA of *Jeotgalicoccus* sp. ATCC 8456 using the primer pair of OleT-NdeI/OleT-*Hin*dIII (Table 1). The gel-cleaned PCR fragment was double digested by *Nde*I and *Hin*dIII and subsequently ligated into the *Nde*I/*Hin*dIII pre-treated pET28b to afford pET28b-*oleT*_*JE*_ (Additional file 8: Figure S7A). For pET28b-*oleT*_*JE*_*-RhFRED*, the genes of *oleT*_*JE*_ and *RhFRED* were first fused by overlap extension PCR [58]. Briefly, the gene encoding RhFRED reductase was amplified from the previously constructed pET28b-*pikC*-*RhFRED*[59,60] with a pair of primers including RhFRED-F and RhFRED-R. The *oleT*_*JE*_ gene was amplified from pET28b-*oleT*_*JE*_, using the primers OleT-F and OleT-RhFRED-OE. Then the two PCR fragments with overlap sequence were mixed, annealed, extended and finally amplified with the OleT-F/RhFRED-R primer pair, giving rise to the fused gene of *oleT*_*JE*_-*RhFRED*. This fusion product was digested by *Nde*I/*Hin*dIII and inserted into the *Nde*I/*Hin*dIII-digested pET28b to generate pET28b-*oleT*_*JE*_-*RhFRED* (Additional file 8: Figure S7B).

Using the genomic DNA of *E. coli* DH5α as template, the two genes encoding Fld and FdR were amplified with the primer pairs of Fld-BamHI-F/Fld-SalI-R and FdR-BamHI-F/FdR-SalI-R, respectively. Next, the *fld* and *fdR* genes were sub-cloned into pACYCDuet-1 and pCDFDuet-1 respectively, resulting in pACYCDuet-*fld* and pCDFDuet-*fdR*. All sub-cloned sequences were confirmed by DNA sequencing at Sangon Biotech, Shanghai.

### Protein overexpression and purification

The *E. coli* BL21(DE3) cells carrying the recombinant expression vector were grown at 37°C for 16 to 20 h in LB medium containing certain selective antibiotics (50 μg·ml^-1^ kanamycin, 34 μg·ml^-1^ chloramphenicol, or 50 μg·ml^-1^ streptomycin), which were used to inoculate (1:100 ratio) Terrific Broth medium containing corresponding antibiotics, thiamine (1 mM), 10% glycerol and a rare salt solution [61]. Cells were grown at 37°C for 3 to 4 h until the optical density at 600 nm (OD_600_) reached 0.6 to 0.8, at which isopropyl-*β*-_D_-thiogalactopyranoside (IPTG, 0.2 mM as final concentration) and *δ*-aminolevulinic acid (0.5 mM, only for P450 expression) were added, followed by 18 h of cultivation at 18°C.

Protein purification was carried out as described elsewhere [61] with slight modifications. Specifically, the cell pellets harvested by centrifugation (5,000 × g, 4°C, 15 min) were stored at -80°C and melted at ambient temperature immediately before use. Then the cell pellets were resuspended in 40 ml of pre-chilled lysis buffer (pH 8.0, 50 mM NaH_2_PO_4_, 300 mM NaCl, 10% glycerol and 10 mM imidazole) through vortexing. Following a sonication step, the cell lysate was centrifuged at 12,000 × g for 30 min to remove the insoluble fraction. To the supernatant, 1 ml of Ni-NTA resin was added and gently mixed at 4°C for 1 h. The slurry was loaded onto an empty column, and washed with approximately 100 ml of wash buffer (pH 8.0, 50 mM NaH_2_PO_4_, 300 mM NaCl, 10% glycerol and 20 mM imidazole) until no proteins were detectable in flow-through. The bound target proteins were eluted with elution buffer (pH 8.0, 50 mM NaH_2_PO_4_, 300 mM NaCl, 10% glycerol and 250 mM imidazole). The eluent was concentrated with an Amicon Ultra centrifugal filter, and buffer exchanged on a PD-10 desalting column. Finally, the desalted purified proteins (Additional file 1: Figure S1) in storage buffer (pH 7.4, 50 mM NaH_2_PO_4_, 10% glycerol) were flash-frozen by liquid nitrogen and stored at -80°C for later use.

### Determination of protein concentration

Following the method described by Omura and Sato [62], the CO-bound reduced difference spectrum (Additional file 2: Figure S2) of each P450 enzyme was recorded on a UV-visible spectrophotometer DU 800 (Beckman Coulter, Fullerton, CA, USA). The functional P450 concentration was calculated using the extinction coefficient (ϵ_450–490_) of 91,000 M^-1^·cm^-1^. The concentration of Fld and FdR from *E. coli* was determined using ϵ_579_ = 4,570 M^-1^·cm^-1^[63] and ϵ_456_ = 7,100 M^-1^·cm^-1^[64], respectively.

### *In vitro* enzymatic assays with purified proteins

The fatty acid decarboxylation assays containing 0.2 to 1.0 μM OleT_JE_ or OleT_JE_-RhFRED, 200 μM fatty acid substrate (from C_8_ to C_20_), 500 μM H_2_O_2_ (for OleT_JE_) or 500 μM NADPH (for OleT_JE_-RhFRED) in 200 μl of storage buffer were carried out at 28°C for 2 h. In the assay to test whether *E. coli* Fld and FdR are able to support the activity of OleT_JE_, 5 μM Fld and 5 μM FdR were mixed with 200 μM myristic acid, 500 μM NADPH and 1 μM OleT_JE_ in 200 μl of storage buffer. To remove spontaneously generated H_2_O_2_, bovine liver catalase was added to the final concentration of 20 U·ml^-1^.

All above described reactions were quenched by addition of 20 μl of 10 M HCl. Heptadecanoic acid was added as internal standard and the mixture was extracted by 200 μl ethyl acetate. Samples were then analyzed by gas chromatography-mass spectroscopy (GC-MS; see below).

### *In vivo* production of *α*-alkenes

The *E. coli fadD* deletion mutant strain XL100 [43] that overproduces fatty acids was selected as the starting host for construction of alkene-producing strains. Plasmids pMSD8 and pMSD15 were gifts from Dr. John Cronan and were used in the strain XL100 to construct fatty acid-overproducing strains. Bacterial cells transformed with certain plasmid(s) (Table 1) were either grown in 50 ml of LB medium or in 50 ml of defined mineral medium [14] containing 3% glucose as the carbon source, supplemented with appropriate selective antibiotics, thiamine (1 mM) and a rare salt solution. All cultivations were performed at 37°C and induced at an OD_600_ of 0.9 to 1.0 with 0.4% arabinose (for pMSD15) followed by 0.2 mM IPTG (for pMSD8, and pET28b-*oleT*_*JE*_ or pET28b-*oleT*_*JE*_-*RhFRED*) after 0.5 h. Next, cells were grown at 28°C (the optimal temperature for OleT_JE_ expression) for an additional 20 h at 220 rpm (in LB medium) or 40 h at 250 rpm (in the defined mineral medium). For analysis of hydrocarbon production, 20 ml of culture with 10 μl heptadecanoic acid added as internal standard was sonicated for 10 min and then thoroughly mixed with an equal volume of chloroform-methanol (2:1, vol/vol). The aqueous-organic mixture was centrifuged (8,000 × g for 15 min) for phase separation. The organic phase was transferred into a clean tube, evaporated under a nitrogen flow, and re-dissolved in 500 μl of n-hexane as the testing sample. Prior to GC-MS analysis, 5 μl eicosane was added as calibration standard. All experiments were repeated two to four times.

### Analytical methods

The GC-MS analytical method for hydrocarbon and fatty acid samples was adapted from Guan *et al.*[65]. The analyses were performed on an Agilent 7890A gas chromatograph equipped with a capillary column HP-INNOWAX (Agilent Technologies, Santa Clara, CA, USA; cross-linked polyethylene glycerol, i.d. 0.25 μm film thickness, 30 m by 0.25 mm) coupled to an Agilent 5975C MSD single quadrupole mass spectrometer operated under electron ionization mode at 70 eV in the scan range of 50 to 500 m/z. The helium flow rate was set to 1 ml·min^-1^. The oven temperature was controlled initially at 40°C for 4 min, then increased at the rate of 10°C per min to 250°C, and held for 15 min. The injecting temperature was set to 280°C with the injection volume of 1 μl under splitless injection conditions. Under these conditions, the previously reported thermal degradations of *α*- and *β*-hydroxy fatty acids to form alkenes (the minor products of OleT_JE_ catalyzed reaction) in the GC inlet [16] were not observed (Additional file 9: Figure S8), making the silylating protection of the hydroxyl group unnecessary if only caring about the *α*-alkene products. To detect *α*- and *β*-hydroxy fatty acids, samples were derivatized with an equal volume of *N, O*-bis(trimethylsilyl)trifluoroacetamide with 1% trimethylchlorosilane at 70°C for 15 min. GC-MS analysis followed the previous protocol developed by Rude *et al.*[16] except for using the Agilent J&W DB-5 MS column (i.d. 0.25 μm film thickness, 50 m by 0.25 mm). During GC-MS analysis, peak identity was determined by comparison of retention time and fragmentation pattern with authentic standard compounds where available and to the National Institute of Standards and Technology, USA mass spectral database. The location of double bond(s) in *α*-olefins was deduced by derivatization with dimethyl disulfide as described previously [66]. Quantification was achieved by comparison of integrated peak areas with calibration curves of authentic standards. The conversion percentages of free fatty acids to corresponding *α*-alkenes were estimated using the equation: [total alkenes]/([total alkenes] + [total free fatty acids]).

## Abbreviations

ACP: acyl carrier protein; CoA: coenzyme A; CYP: cytochrome P450 enzyme; DTT: dithiothreitol; FdR: flavodoxin reductase; Fld: flavodoxin; GC-MS: gas chromatography-mass spectrometry; IPTG: Isopropyl-*β*-_D_-thiogalactopyranoside; LB: lysogeny broth; OD: optical density; PCR: polymerase chain reaction; RhFRED: *Rhodococcus* fusion reductase.

## Competing interests

The authors declare that they have no competing interests.

## Authors’ contributions

SL conceived of the study. YL, CW, XL and SL designed the experiments. YL, JY and WZ performed the experiments including plasmid construction, protein overexpression, purification, characterization and enzymatic assays. YL, CW and WG carried out GC-MS analysis. YL, CW and SL drafted the manuscript. JY, WZ, WG and XL helped to revise the manuscript. All authors read and approved the final manuscript.

## Supplementary Material

Additional file 1: Figure S1 SDS-PAGE analysis of purified **(A)** OleT_JE_ and **(B)** OleT_JE_-RhFRED. M, protein marker. Click here for file

Additional file 2: Figure S2 CO-bound reduced spectra of purified **(A)** OleT_JE_ and **(B)** OleT_JE_-RhFRED. Click here for file

Additional file 3: Figure S3 Protein sequence alignment of CYP152 family members and three selected P450 monooxygenases. Protein sequences were obtained from NCBI protein databases. CYP152A1 (P450_BSβ_ from *Bacillus subtilis*); CYP152A2 (P450_CLA_ from *Clostridium acetobutylicum*); CYP152B1 (P450_SPα_ from *Sphingomonas paucimobilis*); CYP152B2 (from *Azotobacter vinelandii*); CYP152C1 (from *Rhodobacter sphaeroides*); CYP152C2 (from *Rhodobacter sphaeroides*); CYP152D1 (from *Streptomyces scabies*); CYP152E1 (from *Cyanothece* sp. CCY0110); CYP107L1 (P450_PikC_ from *Streptomyces venezuelae*); CYP102A1 (P450_BM3_ from *Bacillus megaterium*); CYP101A1 (P450_cam_ from *Pseudomonas putida*). Conserved amino acid residues are shaded. The Arg and Pro absolutely conserved in CYP152 family are marked by asterisks. Click here for file

Additional file 4: Figure S4 GC-MS analysis of the organic extract of the YL7 culture in LB broth. Eicosane and heptadecanoic acid are served as calibration standard (CS) and internal standard (IS), respectively. Click here for file

Additional file 5: Figure S5 The dry cell weight of the YL5-8 cultures using LB broth. Click here for file

Additional file 6: Figure S6 Production profile of free fatty acids by the strain XL100/(pMSD8 + pMSD15). Click here for file

Additional file 7: Table S1 Primers used in this study. Click here for file

Additional file 8: Figure S7 Plasmid maps for **(A)** pET28b-*oleT*_*JE*_ and **(B)** pET28b-*oleT*_*JE*_-*RhFRED* expression vectors. Click here for file

Additional file 9: Figure S8 GC-MS analysis of the *α*- and *β*-hydroxy myristic acid authentic standards on HP-INNOWAX capillary column. **(A)** Without derivatization, *α*-hydroxy myristic acid was unseen due to its high boiling point. Importantly, no thermally degraded terminal olefin (1-tridecene) was observed in the dashed box. **(B)** Without derivatization, *β*-hydroxy myristic acid was unseen due to its high boiling point. Again, no thermally degraded terminal olefin (1-tridecene) was observed in the dashed box. **(C)** The decarboxylation reaction of myristic acid (2 h) catalyzed by OleT_JE_. The peak shown in the dashed box corresponds to 1-tridecene. Click here for file
