# Supplementary figures and images for: Hydrogen peroxide-independent production of α-alkenes by OleTJE P450 fatty acid decarboxylase
Source: Biotechnol Biofuels. 2014 Feb 24;7:28. doi: 10.1186/1754-6834-7-28 (PMC3937522; doi:10.1186/1754-6834-7-28)

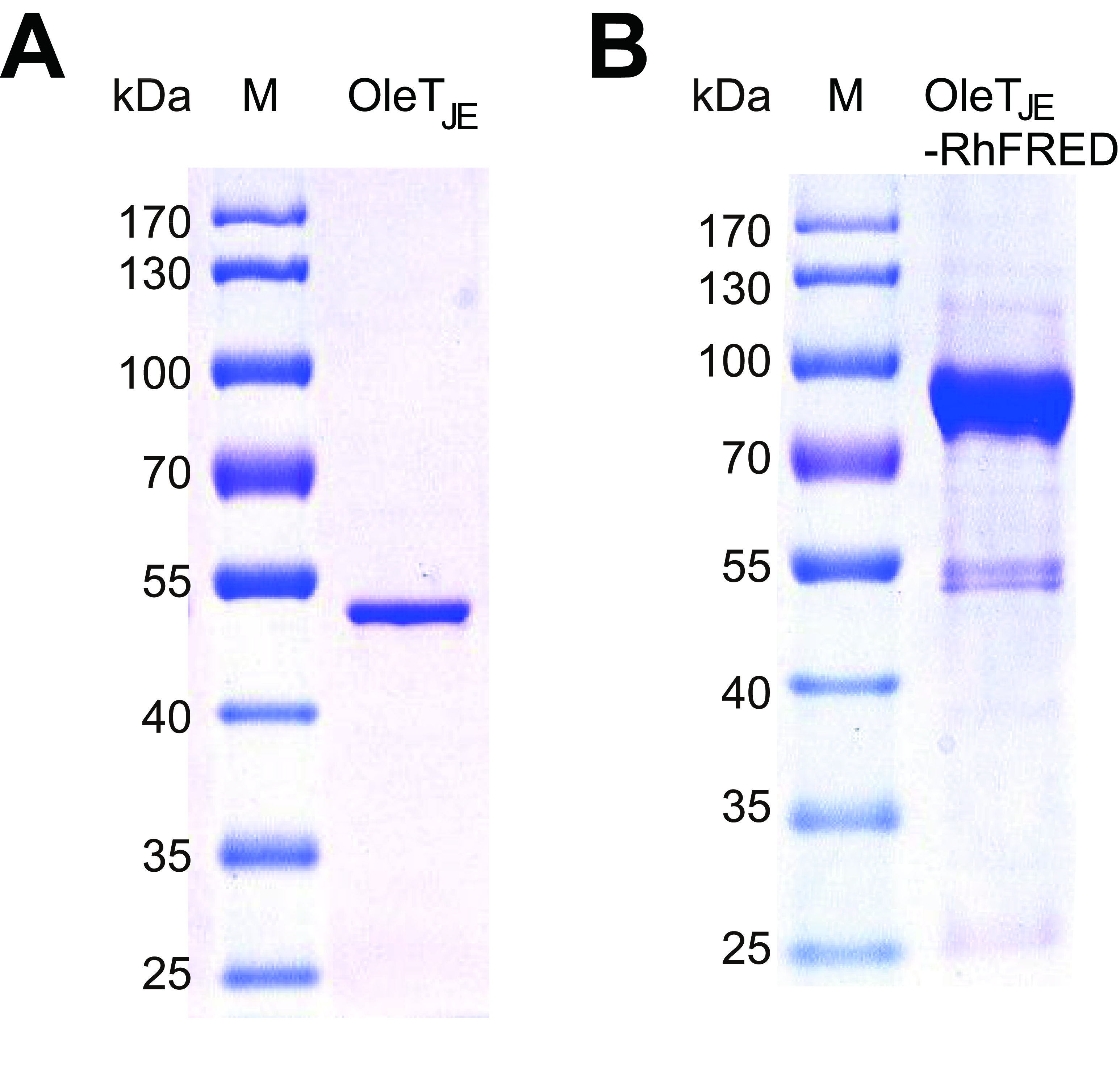

Supplement: Additional file 1: Figure S1 — SDS-PAGE analysis of purified (A) OleTJE and (B) OleTJE-RhFRED. M, protein marker. [file 1754-6834-7-28-S1.tiff]

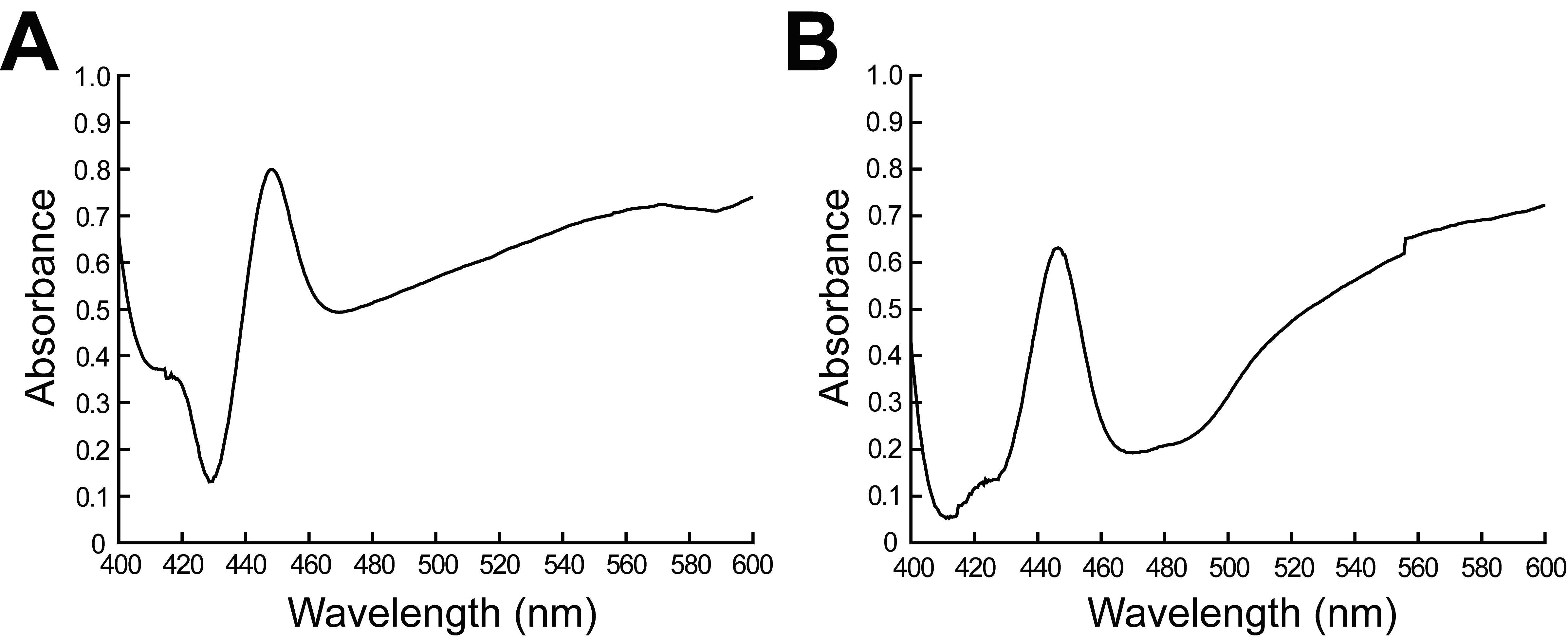

Supplement: Additional file 2: Figure S2 — CO-bound reduced spectra of purified (A) OleTJE and (B) OleTJE-RhFRED. [file 1754-6834-7-28-S2.tiff]

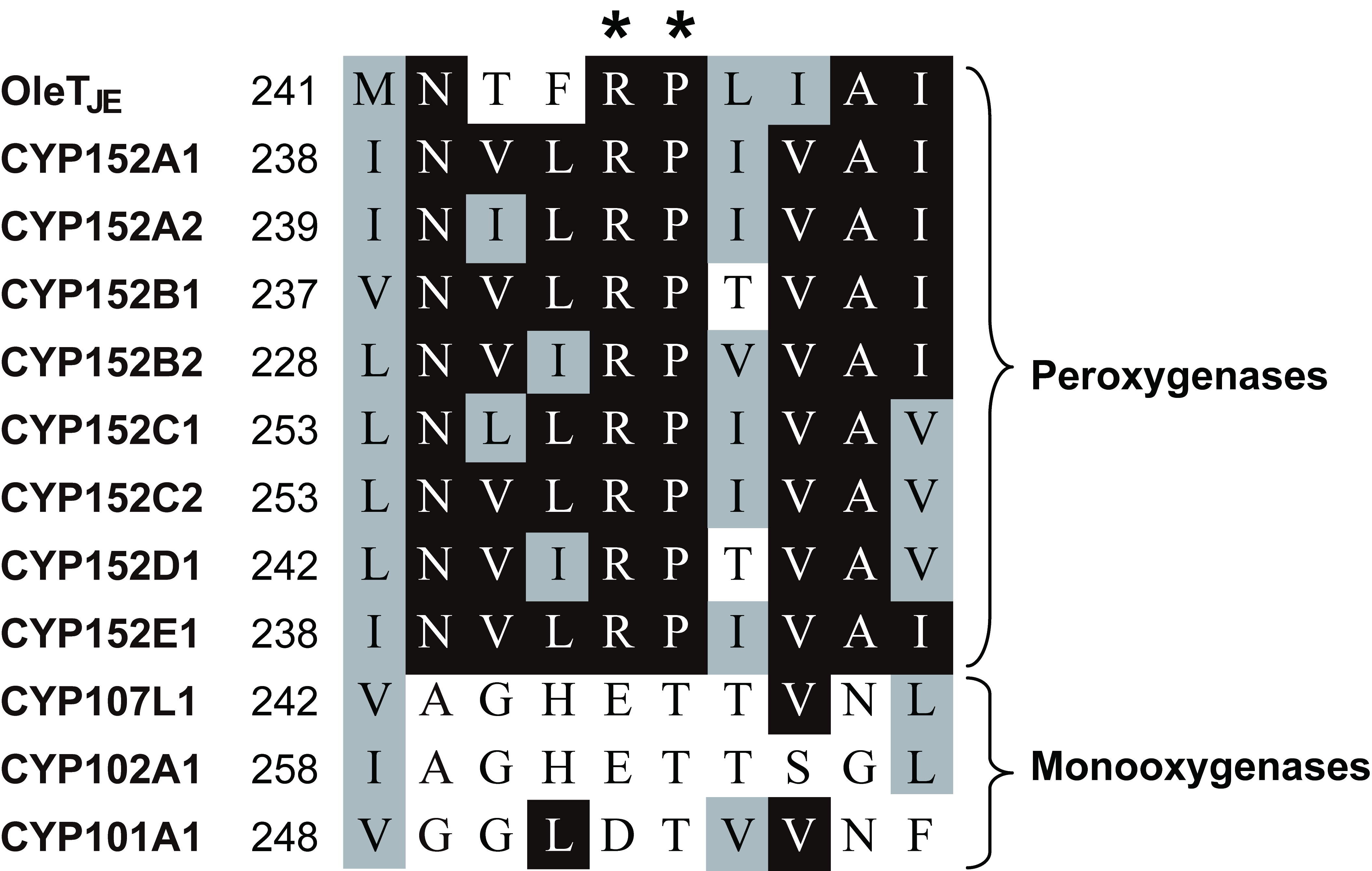

Supplement: Additional file 3: Figure S3 — Protein sequence alignment of CYP152 family members and three selected P450 monooxygenases. Protein sequences were obtained from NCBI protein databases. CYP152A1 (P450BSβ from Bacillus subtilis); CYP152A2 (P450CLA from Clostridium acetobutylicum); CYP152B1 (P450SPα from Sphingomonas paucimobilis); CYP152B2 (from Azotobacter vinelandii); CYP152C1 (from Rhodobacter sphaeroides); CYP152C2 (from Rhodobacter sphaeroides); CYP152D1 (from Streptomyces scabies); CYP152E1 (from Cyanothece sp. CCY0110); CYP107L1 (P450PikC from Streptomyces venezuelae); CYP102A1 (P450BM3 from Bacillus megaterium); CYP101A1 (P450cam from Pseudomonas putida). Conserved amino acid residues are shaded. The Arg and Pro absolutely conserved in CYP152 family are marked by asterisks. [file 1754-6834-7-28-S3.tiff]

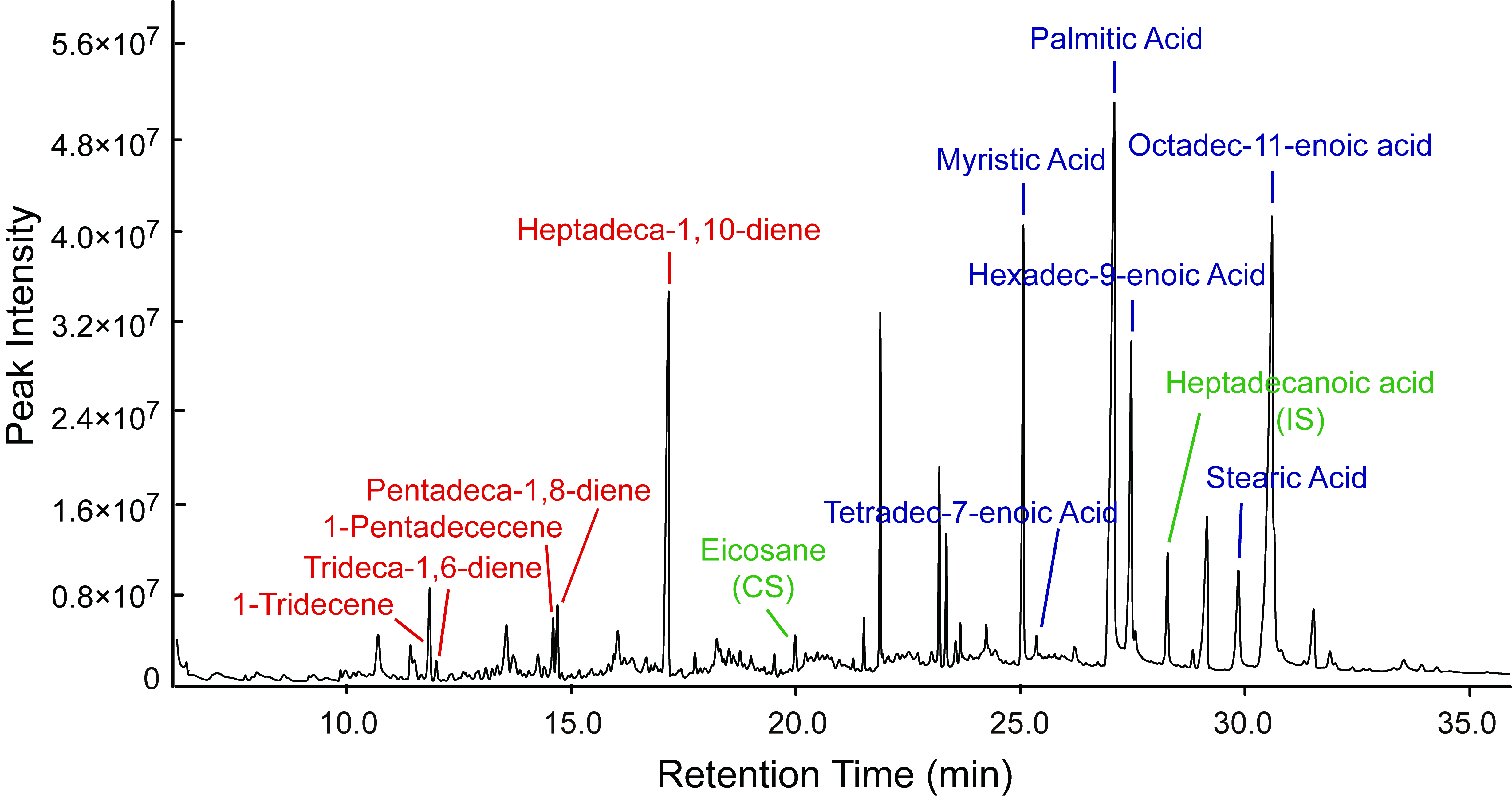

Supplement: Additional file 4: Figure S4 — GC-MS analysis of the organic extract of the YL7 culture in LB broth. Eicosane and heptadecanoic acid are served as calibration standard (CS) and internal standard (IS), respectively. [file 1754-6834-7-28-S4.tiff]

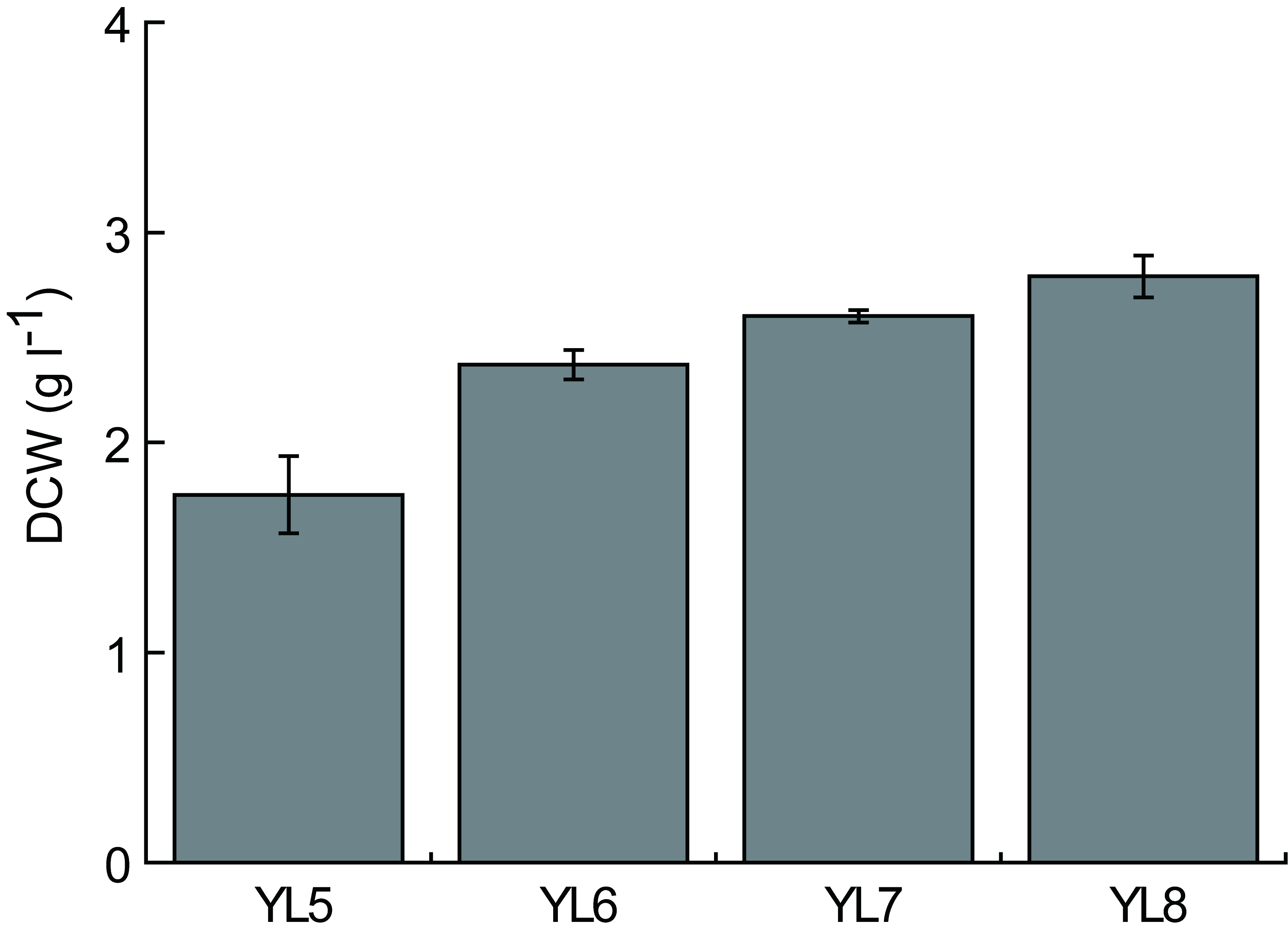

Supplement: Additional file 5: Figure S5 — The dry cell weight of the YL5-8 cultures using LB broth. [file 1754-6834-7-28-S5.tiff]

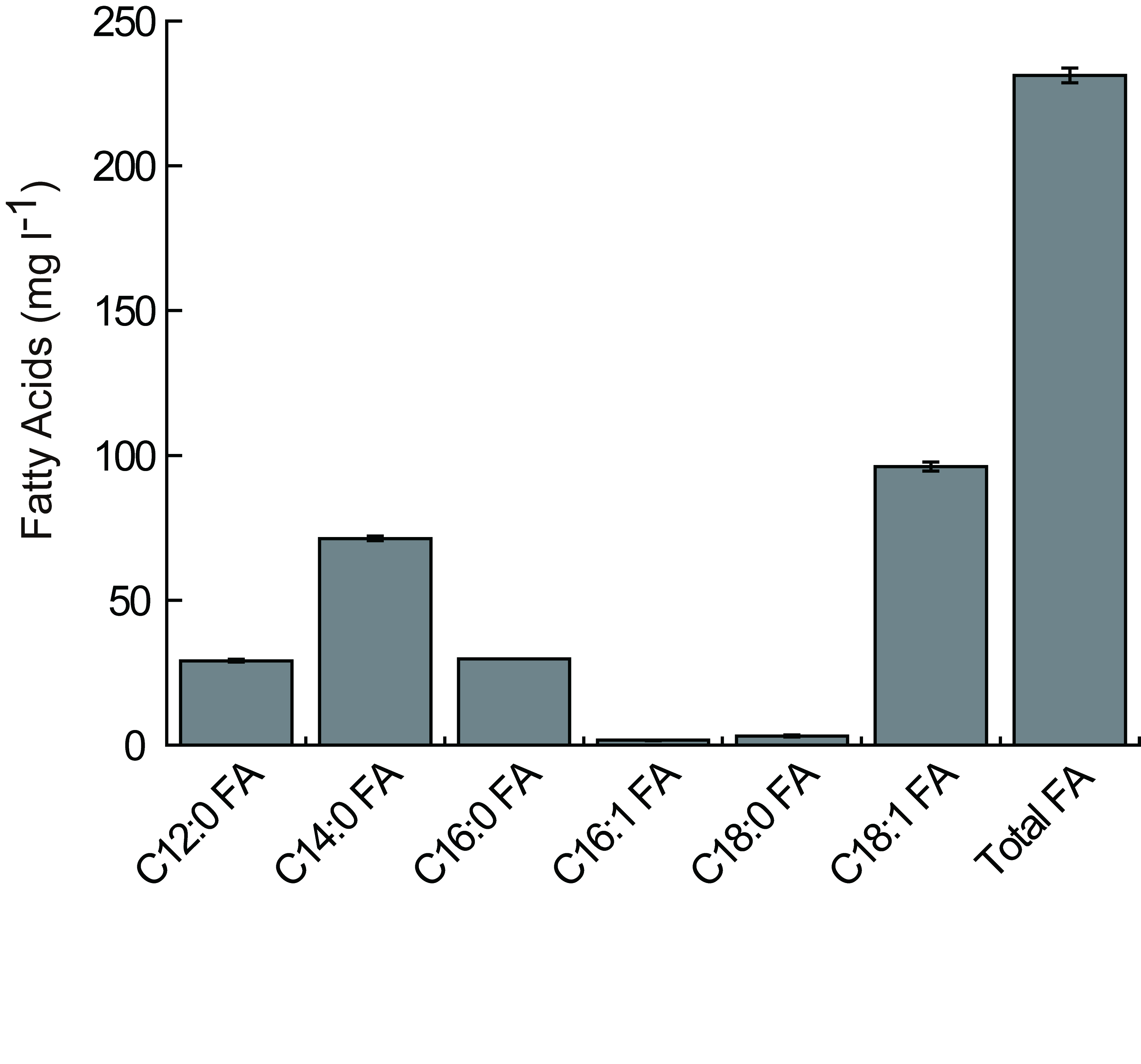

Supplement: Additional file 6: Figure S6 — Production profile of free fatty acids by the strain XL100/(pMSD8 + pMSD15). [file 1754-6834-7-28-S6.tiff]

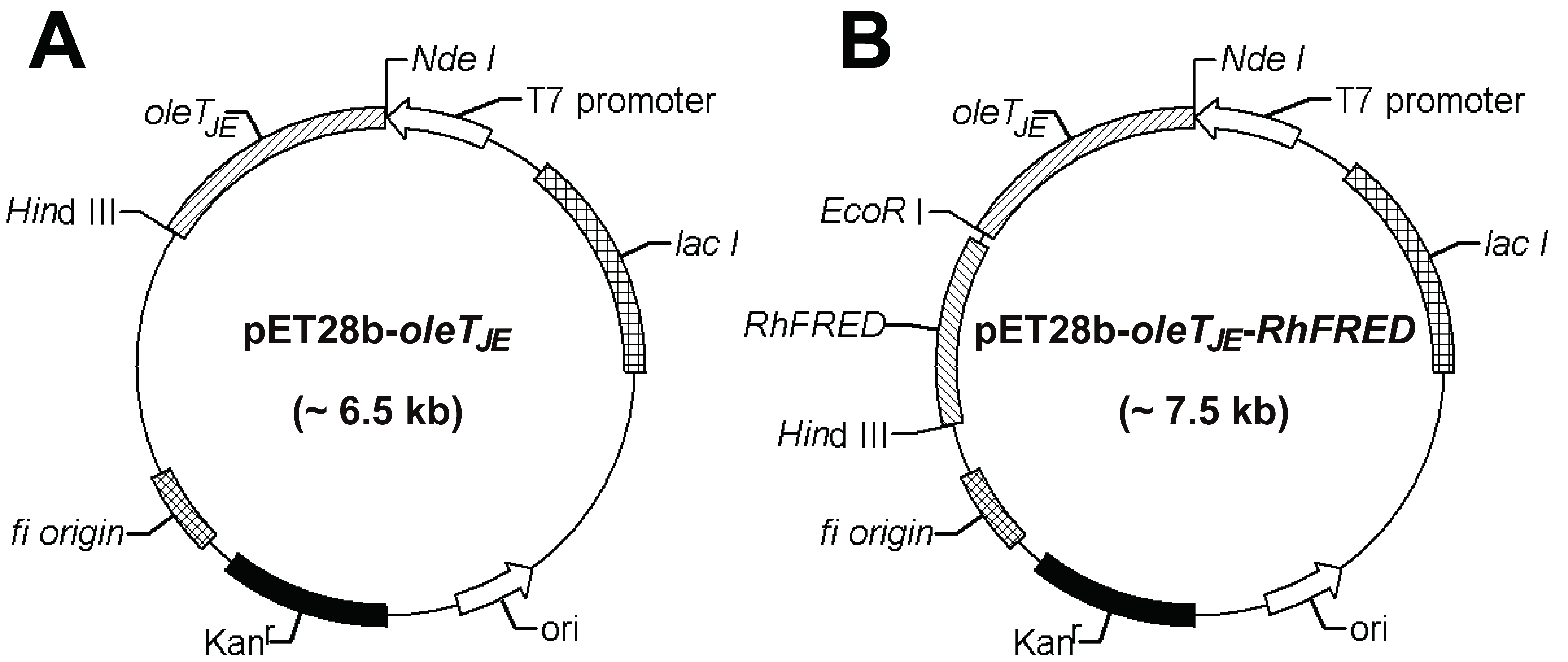

Supplement: Additional file 8: Figure S7 — Plasmid maps for (A) pET28b-oleT JE and (B) pET28b-oleT JE -RhFRED expression vectors. [file 1754-6834-7-28-S8.tiff]

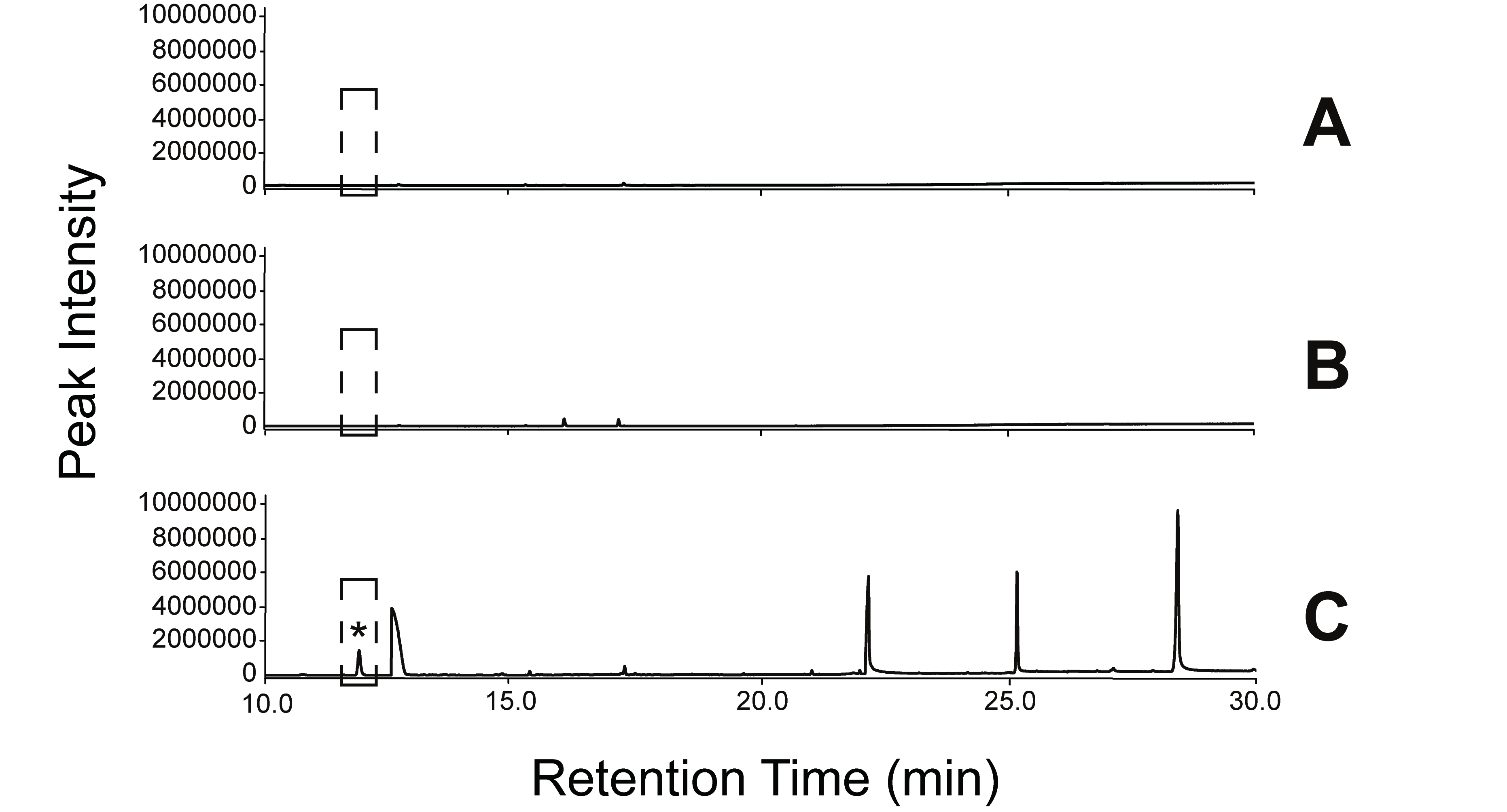

Supplement: Additional file 9: Figure S8 — GC-MS analysis of the α- and β-hydroxy myristic acid authentic standards on HP-INNOWAX capillary column. (A) Without derivatization, α-hydroxy myristic acid was unseen due to its high boiling point. Importantly, no thermally degraded terminal olefin (1-tridecene) was observed in the dashed box. (B) Without derivatization, β-hydroxy myristic acid was unseen due to its high boiling point. Again, no thermally degraded terminal olefin (1-tridecene) was observed in the dashed box. (C) The decarboxylation reaction of myristic acid (2 h) catalyzed by OleTJE. The peak shown in the dashed box corresponds to 1-tridecene. [file 1754-6834-7-28-S9.tiff]
