# Supplementary material for: Hydrogen peroxide-independent production of α-alkenes by OleTJE P450 fatty acid decarboxylase
Source: Biotechnol Biofuels. 2014 Feb 24;7:28. doi: 10.1186/1754-6834-7-28 (PMC3937522; doi:10.1186/1754-6834-7-28)
Supplement: Additional file 7: Table S1 — Primers used in this study. [file 1754-6834-7-28-S7.pdf]

Table S1. Primers used in this study

**Primer name**	**Primer sequence** (5'→3')	**Function of underlined bases**
OleT-NdeI	GTATTTCCATATGGCAACACTTAAGAGGGATAAG	*Nde*I site
OleT-HindIII	CAATGAAGCTTTTATGTTCTGTCTACAACTTCGC	*Hin*dIII site
OleT-F	GCGCCATATGATGGCAACACTTAAGAGGGA	*Nde*I site
OleT-RhFRED-OE	TGACCGGTTGATGCCGGTGCAGCACGAATTCTGTTCTGTCTACAACTTCGCGAACA	overlap sequence for gene fusion
RhFRED-F	GAATTCGTGCTGCACCGGCATCAACCGGTCA	*Eco*RI site
RhFRED-R	TCCCAAGCTTTCAGAGTCGCAGGGCCAGCC	*Hin*dIII site
FdR-BamHI-F	CGCGGGATCCGATGGCTGATTGGGTAACA	*Bam*HI site
FdR-SalI-R	CAACGTCGACTTACCAGTAATGCTCCGC	*Sal*I site
Fld- BamHI-F	CGCGGGATCCT-ATGGCTATCACTGGCATC	*Bam*HI site
Fld-SalI-R	CGCGGTCGACTCAGGCATTGAGAATTTC	*Sal*I site
